# Enhancing population-level research among people who inject drugs: a validation and retrospective cohort study using health administrative data in Ontario, Canada

**DOI:** 10.23889/ijpds.v9i5.2628

**Published:** 2024-09-18

**Authors:** Zoë R Greenwald, Jordan J Feld, Dan Werb, Peter C Austin, Samantha Drover, Daniel Fridman, Ahmed Bayoumi, Tara Gomes, Claire E Kendall, Lauren Lapointe-Shaw, Ayden I Scheim, Sofia R Bartlett, Eric I Benchimol, Zachary Bouck, Christina Greenaway, Naveed Z Janjua, Pamela Leece, William WL Wong, Beate Sander, Jeffrey C Kwong

**Affiliations:** 1 University of Toronto; 2 University Health Network; 3 ICES; 4 Bruyère Research Institute; 5 Centre on Drug Policy Evaluation, St. Michael's Hospital; 6 Drexel University; 7 British Columbia Centre for Disease Control; 8 Division of Infectious Diseases, Jewish General Hospital; 9 Public Health Ontario; 10 University of Waterloo

## Objective

Health administrative data can support population-level research among people who inject drugs (PWID), however these data sources remain underutilized due to the difficulty in identifying drug use in routinely collected data. We validated case-ascertainment algorithms to identify PWID and tested their application in a hepatitis C (HCV) cohort in Ontario, Canada.

## Approach

We conducted a validation study using reference standard cohorts of PWID recruited via community-based studies and population controls linked to health administrative data in Ontario, Canada (1992-2020). Tested case-ascertainment algorithms included combinations of hospitalizations/emergency department (ED) visits for drug use/poisoning, physician visits for drug use, opioid agonist treatment (OAT), or injecting-related infections. Sensitivity and specificity were estimated for lifetime history and recent injecting (past 1-5 years). We applied a high-performing algorithm among all Ontarians with laboratory-confirmed HCV between 1999-2018, to identify a sub-cohort of PWID with HCV.

## Results

An algorithm including ≥1 hospitalization/ED visit or ≥1 physician visit for drug use or ≥1 OAT record had high accuracy for identifying IDU history (91.6% sensitivity, 94.2% specificity) and recent IDU (using 3 years lookback: 80.4% sensitivity, 99% specificity). When applied to a provincial cohort of 112,947 Ontarians diagnosed with HCV, this algorithm estimated 46% (N=52,248) had a history of IDU, of whom 52% (N=27,246) had an indication of recent IDU (within the past 3 years).

## Conclusion

The methods developed in this study can enhance the capacity of population-level health research among people who inject drugs and support applied public health interventions towards hepatitis C elimination.

